# Mild Abdominal Trauma Complicated With Bowel Perforation in Patient With Crohn’s Disease: Clinical and Intraoperative Findings

**DOI:** 10.7759/cureus.21977

**Published:** 2022-02-07

**Authors:** Ayman M Babiker, Abdullah Hamad A Alkharraz, Hamad A Yusef Alsaeed, Adi Abdulaziz R Aldubaiyan

**Affiliations:** 1 Surgical Oncology, King Fahad Medical City, Riyadh, SAU; 2 Surgery, Qassim University, Unaizah, SAU; 3 Medicine, Qassim University, Unaizah, SAU

**Keywords:** post operative (po), junction between the cecum and ascending colon (jbcac), low intensity blunt trauma (libt), crohn’s disease (cd), computed tomography (ct )

## Abstract

Crohn’s disease (CD) is a chronic inflammatory disease and is an autoimmune disorder. Due to its remission and relapse cycle, the integrity of the intestinal wall becomes compromised in patients with inflammatory bowel disease. This increases susceptibility to bowel perforation following mechanical injury, especially in patients using immunosuppressive therapy. However, few reports discuss the severity of injury after abdominal trauma in Crohn’s patients and how much mild abdominal blunt trauma in such patients can be different in the presentation clinically in correlation with the severity of the intraoperative findings. We report a case of ascending colon perforation secondary to mild abdominal blunt trauma in a patient with CD.

## Introduction

Crohn’s disease (CD) is a chronic relapsing and remitting inflammatory disease that mainly affects the GI tract and has extraintestinal complications [1]. The disease prevalence and incidence are higher in developed countries [2].

The disease commonly presents with diarrhea, weight loss, and abdominal pain that is usually located in the right lower quadrant. There are less common symptoms like fatigue, rectal bleeding, or bleeding with diarrhea, especially if the colon is affected. There are extraintestinal manifestations like erythema nodosum, periarticular large joint arthritis, primary sclerosing cholangitis, and axial arthropathies, but the last two are independent of the disease flares and activity [2].

The diagnosis of CD relay on clinical, histological, and morphological data [3]. Colonoscopy is the gold standard diagnostic tool used in Crohn’s disease. 

CD has multiple complications, like abscesses, fistulas, and fissures [4]. In addition, due to its relapsing and remitting nature, the integrity of the intestinal wall becomes compromised and predisposes to injury with mild blunt abdominal trauma. We report a case representing this phenomenon.

## Case presentation

**Figure 1:**
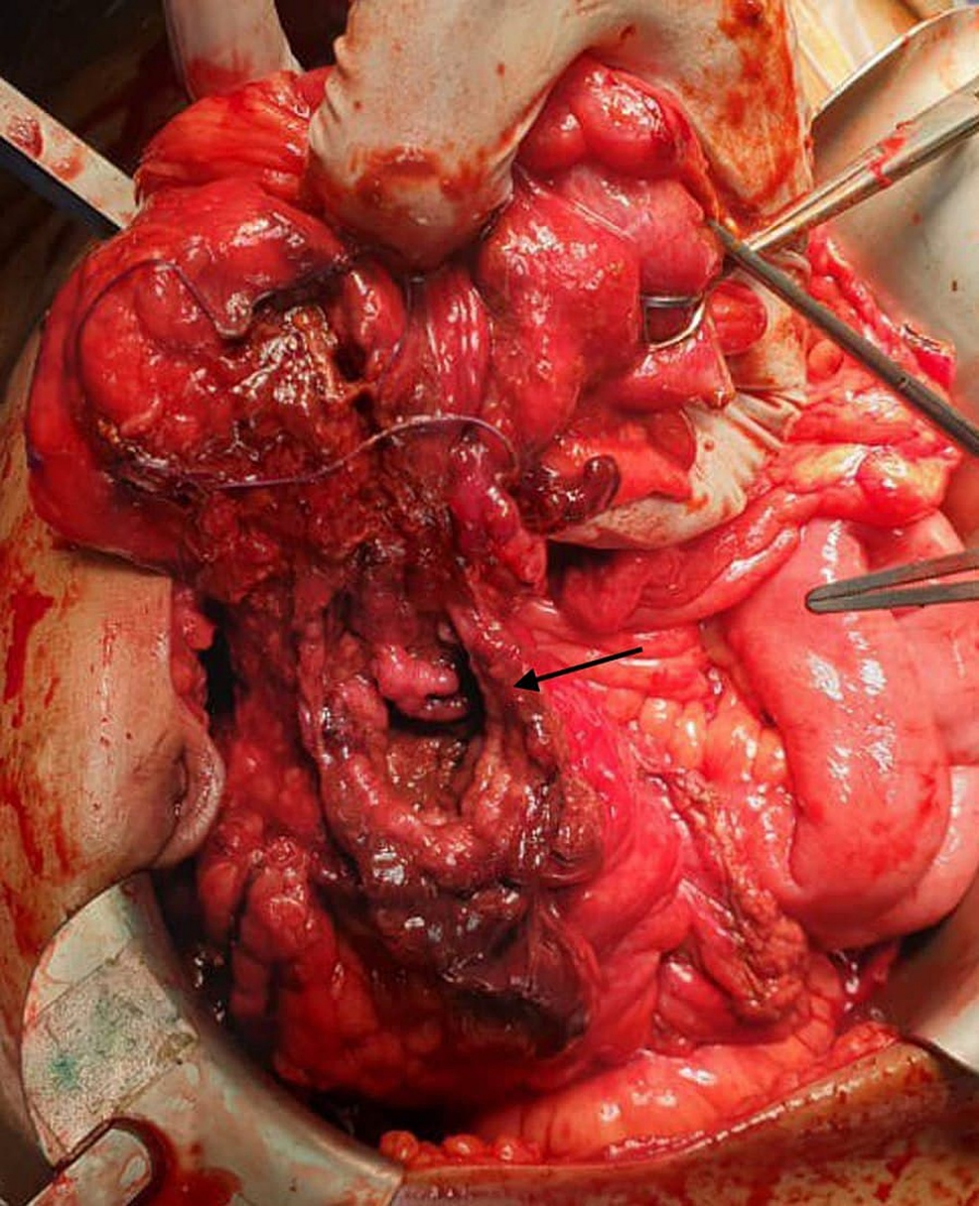
Perforation to the ascending colon.

**Figure 2:**
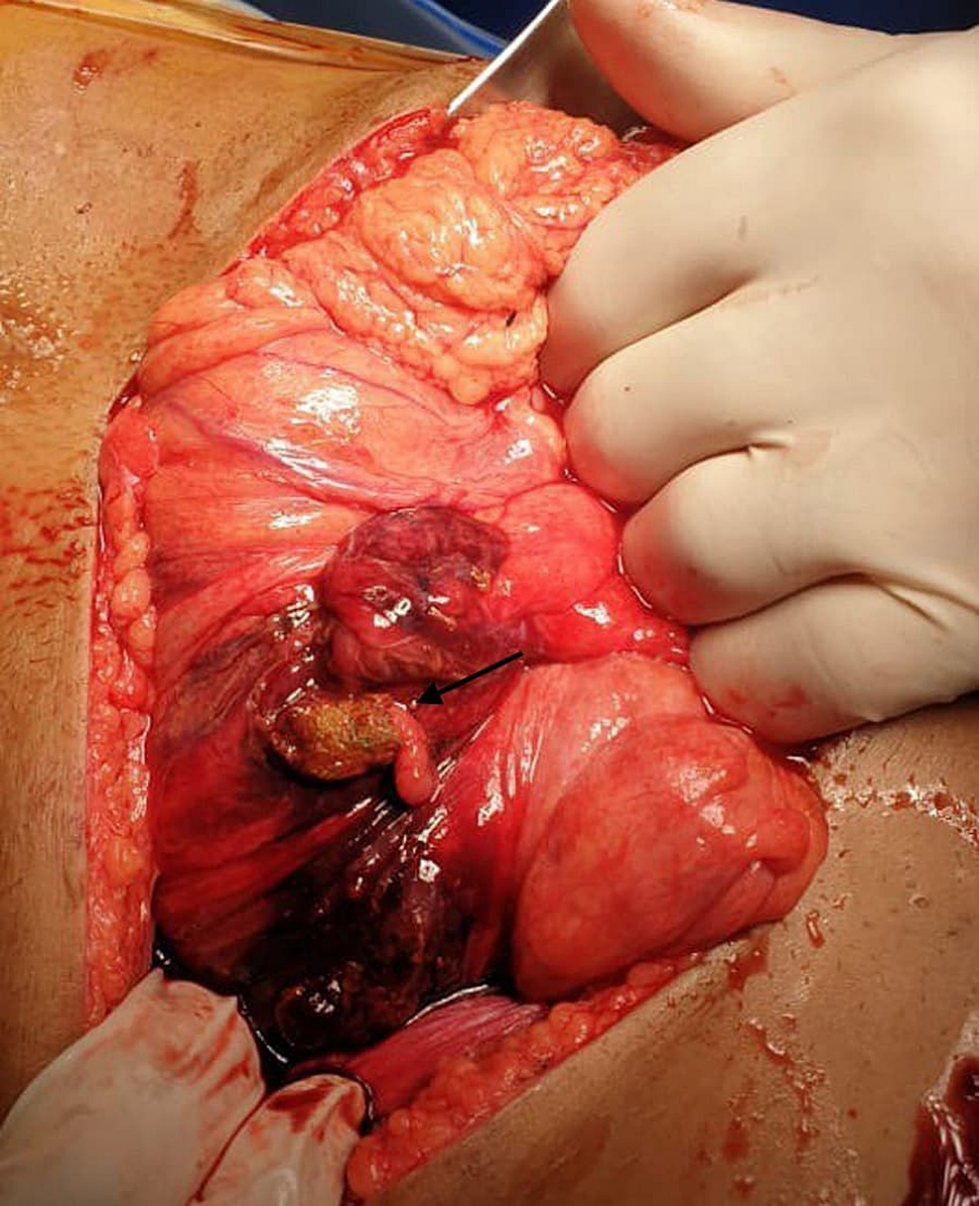
Ascending colon perforation with fecal contamination.

On October 5, 2019, a 25-year-old male, known case of CD, presented to the ER after a motor vehicle accident with low-intensity blunt trauma (LIBT), complaining of only abdominal pain. He had a history of transient loss of consciousness and vomiting following the accident. The patient was vitally stable, blood pressure 132/78 mmHg, pulse 69, oxygen saturation 99%, respiratory rate 18, temperature 36.8, and neurological status was intact. On clinical examination, he was conscious, alert, and oriented. There was equal air entry in both sides of the lungs with no tenderness on the chest. The abdomen was not distended with moderate tenderness all over and guarding to palpation. CT of the brain, spine, chest, abdomen, and pelvis showed free intraperitoneal air and mild free fluid in the pelvic. Given these findings, an urgent exploratory laparotomy was indicated, showing evidence of injury to the junction between the cecum and ascending colon (JBCAC) with almost transection involving 90% of the circumference (Figure 1) with gross contamination of peritoneal cavity with stool (Figure 2), minimal hemoperitoneum, and hematoma. Additionally, a small mesenteric tear was noted. Then ileocecal resection was completed with an end loop ileostomy. The patient tolerated the procedure and was extubated in stable condition. After 11 days post-operative (PO), the patient was discharged with regular outpatient clinic follow-up, with no active complaint. On October 15, 2019, ileostomy revision was indicated with ileocolic anastomosis with no complications. After six days PO, the patient was discharged in stable condition. 

## Discussion

CD is a chronic inflammatory disorder that can involve any segment of the digestive tract. Data reports that CD can affect 1 to 6/100,000 inhabitants [5]. There is no clear cause of CD, but genetics and environmental factors are suspected in its etiology. Intestinal perforation is one of the most common inflammatory bowel disease complications. Up to 40% of patients with CD can develop intestinal perforation after 10 years of the disease course. Other complications include fistula and abscess [3]. A study showed that surgery is necessary for up to 80% of patients with CD. As per European guidelines, surgical options must be discussed with patients, and anti-TNF therapy is considered for them [6].

There is a lack of studies in the literature on bowel perforation in patients with inflammatory bowel disease following mild abdominal trauma. There is a similar case report of a 20-year-old male patient with ileocolonic CD, presented to the emergency department following an abdominal hit in football practice. He had signs of irritation of the peritoneum, but due to his stability, a CT abdomen was done, which demonstrated pneumoperitoneum, free fluid, and wall thickening. Due to these findings, an urgent laparotomy was indicated, and a 2-cm perforation in the hepatic angle was found intra-operatively, which indicated a right hemicolectomy to be performed. This patient developed a surgical-site infection and intra-abdominal collection afterward and was discharged on the 30-day PO [7].

Another case report found is on a 22-year-old male with no significant medical history. He was admitted after a road traffic accident. He underwent abdominal CT, which revealed pelvis fractures, abnormal wall of the terminal ileum, peritoneal effusion within the pelvis, mesenteric nodes, and extra-luminal gas within an area of mesenteric inflammation. Based on these features, it was decided that laparoscopic assessment was necessary, which eventually led to the decision of making an ileocecal resection and anastomosis. Diagnosis of CD was made based on the histopathology report, and the patient was discharged on day 9 PO without adverse events [8]. 

## Conclusions

Intraoperative findings in patients with CD on immunosuppressive therapy presenting with LIBT can be severe and life-threatening, which may not reflect the initial clinical presentation and could be disproportional to it. Therefore, a low threshold to urgent abdominal imaging evaluation is needed to determine the severity and planning for surgery if indicated.

## References

[1] Baumgart DC, Sandborn WJ (2012). Crohn’s disease. Lancet.

[2] Torres J, Mehandru S, Colombel JF, Peyrin-Biroulet L (2017). Crohn’s disease. Lancet.

[3] Cosnes J, Cattan S, Blain A, Beaugerie L, Carbonnel F, Parc R, Gendre JP (2002). Long-term evolution of disease behavior of Crohn's disease. Inflamm Bowel Dis.

[4] Feuerstein JD, Cheifetz AS (2017). Crohn disease: epidemiology, diagnosis, and management. Mayo Clin Proc.

[5] Loftus EV Jr (2004). Clinical epidemiology of inflammatory bowel disease: Incidence, prevalence, and environmental influences. Gastroenterology.

[6] Cummings JR, Keshav S, Travis SP (2008). Medical management of Crohn's disease. BMJ.

[7] Pérez-Jiménez A, de la Serna S, Palomar J, Sanz-Ortega G, Torres AJ (2020). Mild abdominal trauma in patients with Crohn’s disease: greater susceptibility to colon perforation?. Cirugía Española (English Ed).

[8] Wagner M, Lefevre JH, Royer B, Svrcek M, Pradel C, Tiret E (2012). Internal fistula leakage due to a road traffic accident: a fortuitous diagnosis of Crohn's disease. J Crohns Colitis.

